# Occupationally Acquired *Salmonella* I 4,12:i:1,2 Infection in a Phlebotomist — Minnesota, January 2013

**Published:** 2013-06-28

**Authors:** Kirk E. Smith, Richard Danila, Joni Scheftel, Heather Fowler, Amy Westbrook, Ginette Dobbins, Mary J. Choi

**Affiliations:** Minnesota Dept of Health; EIS Officer, CDC

On January 25, 2013, the Minnesota Department of Health (MDH) was notified of two clinical cases of *Salmonella* I 4,12:i:1,2 infection with isolates that had indistinguishable pulsed-field gel electrophoresis (PFGE) patterns. Illness onset dates were January 3 and January 9, 2013. Patients A and B were hospitalized at the same hospital during January 12–15 for dehydration. Investigations indicated that these cases were part of a multistate outbreak associated with frozen mice purchased to feed snakes.

On January 25, the MDH Public Health Laboratory isolated *Salmonella* I 4,12:i:1,2 with an indistinguishable PFGE pattern from a third Minnesota resident, patient C. Patient C denied contact with frozen feeder mice or snakes, but was employed as a phlebotomist at the hospital where the two infected patients were hospitalized. Protocol at the hospital requires that each phlebotomist use a hand-held sample tracking device to scan the identification band of each patient from whom blood is drawn. Accessing these records, the infection prevention specialist at the hospital found that patient C drew blood from patient A on January 13 and from patients A and B on January 14, which was 3 days before onset of patient C’s symptoms on January 17. Patient C reported use of gloves while drawing blood.

In the absence of specific evidence for any other risk factor for *Salmonella* I 4,12:i:1,2 infection and considering the temporal relationship between exposure and symptom onset, occupational person-to-person contact with patients A or B likely was the source of patient C’s infection. *Salmonella* transmission from infected patients to health-care workers, although rare, has been reported (1). This investigation documents the first reported case of occupationally acquired *Salmonella* infection in a phlebotomist and underscores the personal risk that health-care workers face when caring for patients. Health-care workers from all disciplines must remain vigilant in protecting themselves from occupationally acquired infections through the use of proven strategies (e.g., regular disinfection of patient-care equipment, hand hygiene, and correct use of personal protective equipment) (2).
